# A three‐lncRNA signature of pretreatment biopsies predicts pathological response and outcome in esophageal squamous cell carcinoma with neoadjuvant chemoradiotherapy

**DOI:** 10.1002/ctm2.156

**Published:** 2020-08-26

**Authors:** Chaoqi Zhang, Zhihui Zhang, Guochao Zhang, Liyan Xue, Haijun Yang, Yuejun Luo, Xiaoli Zheng, Yonglei Zhang, Yufen Yuan, Ruixue Lei, Zhaoyang Yang, Bo Zheng, Zhen Zhang, Le Wang, Yun Che, Sihui Wang, Feng Wang, Lingling Fang, Qingpeng Zeng, Jiagen Li, Shugeng Gao, Qi Xue, Nan Sun, Jie He

**Affiliations:** ^1^ Department of Thoracic Surgery National Cancer Center National Clinical Research Center for Cancer Cancer Hospital Chinese Academy of Medical Sciences and Peking Union Medical College Beijing China; ^2^ Department of Pathology National Cancer Center National Clinical Research Center for Cancer Cancer Hospital Chinese Academy of Medical Sciences and Peking Union Medical College Beijing China; ^3^ Department of Pathology Anyang Cancer Hospital The Fourth Affiliated Hospital of Henan University of Science and Technology Anyang Henan China; ^4^ Department of radiotherapy The Affiliated Cancer hospital of Zhengzhou University Zhengzhou Henan China; ^5^ Department of General Surgery The Affiliated Cancer Hospital of Zhengzhou University Zhengzhou Henan China; ^6^ Biotherapy Center The First Affiliated Hospital of Zhengzhou University Zhengzhou Henan China; ^7^ Department of Otology The First Affiliated Hospital of Zhengzhou University Zhengzhou Henan China

**Keywords:** esophageal squamous cell carcinoma, lncRNAs, neoadjuvant chemoradiotherapy, pathologically complete response, individualized medicine

## Abstract

**Background:**

Current strategies are insufficient to predict pathologically complete response (pCR) for esophageal squamous cell carcinomas (ESCCs) before treatment. Here, we aim to develop a novel long noncoding RNA (lncRNA) signature for pCR and outcome prediction of ESCCs through a multicenter analysis for a Chinese population.

**Methods:**

Differentially expressed lncRNAs (DELs) between pCRs and less than pCR (<pCR) in the pretreated cancer biopsies were identified from 28 cases in Guangzhou cohort and verified from 30 cases in Beijing discovery cohort. Then a prediction model was built through Fisher's linear discriminant analysis (FLDA) of 67 cases in Beijing training cohort. Then an internal cohort and an integrated external cohort (Zhengzhou and Anyang cohorts) were used to validate the predictive accuracy. The prognostic value of this signature was also evaluated.

**Results:**

Twelve DELs were identified from Guangzhou cohort and six lncRNAs were verified. Then, a classifier of three lncRNAs (SCAT1, PRKAG2‐AS1, and FLG‐AS1) was established and achieved a high accuracy with an area under the receiver operating characteristic curve (AUC) of 0.952 in the training cohort, which was well validated in the internal validation cohort and external cohort with the AUCs of 0.856 and 0.817, respectively. Furthermore, the predictive score was identified as the only independent predictor for pCR. Patients with high discriminant score showed a significantly longer overall and relapse‐free survival (*P *< .05).

**Conclusions:**

We developed the first and applicable three‐lncRNA signature of pCR and outcome prediction, which is robust and reproducible in multicenter cohorts for ESCCs with nCRT.

## Graphical abstract

This study revealed the first lncRNA signature that showed powerful predictive value for pathological response in ESCCs with nCRT; The three‐lncRNA signature was the first molecular model to show robust prognostic accuracy in patients with ESCC undergoing nCRT; and the number of cases from multi‐institutions enrolled in this project was considerably larger than any of the previous studiesfruit.

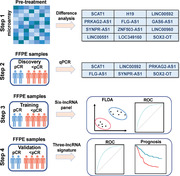

**Abbreviations**
< pCR: less than pCR
18F‐FDG: 18F‐fluoro‐2‐deoxy‐D‐glucose
AUC: area under the curve
CI: confidence interval
DELs: differentially expressed lncRNAs
EAC: esophageal adenocarcinoma
EC: esophageal cancer
ESCC: esophageal squamous cell carcinoma
EUS: endoscopic ultrasonography
FFPE: formalin‐fixed paraffin‐embedded
FLDA: Fisher's linear discriminant analysis
GEO: Gene Expression Omnibus
lncRNAs: long non‐coding RNAs
miRNAs: microRNAs
mRNAs: messenger RNAs
NCC: National Cancer Center
nCRT: neoadjuvant chemoradiotherapy
nRNAs: non‐coding RNAs
OS: overall survival
pCR: pathologically complete response
PET(‐CT): positron emission tomography with or without computed tomography
qPCR: real‐time quantitative polymerase chain reaction
RFS: relapse free survival
RMA: Robust Multiarray Average
ROC: receiver operating characteristic
SVM: support vector machine.

## 1 INTRODUCTION

Esophageal cancer (EC) is the seventh most common cancer and the sixth leading cause of cancer related mortality in the world, with about 572 000 new cases and 509 000 deaths occurred annually worldwide.^1^ Esophageal adenocarcinoma (EAC) and esophageal squamous cell carcinoma (ESCC) are the two major histological types.^2^ In China, ESCC is the predominant tumor type and accounts for >90% cases of EC, where it bears almost half of the global burden.^3^ With high recurrence rates and poor prognosis after surgery, ESCC is consistently regarded as a highly aggressive malignancy.^4^ The amplification of neoadjuvant chemoradiotherapy (nCRT) followed by surgery for locally advanced ESCC has improved survival compared to resection alone with the 5‐year overall survival varying from 47% to 60%, and this multimodality protocol has been recommended as the guidelines of ESCC management.^5^, ^6^, ^7^, ^8^ In fact, the outcomes of patients with ESCC who undergo nCRT are heterogeneous. Only about one‐third patients achieved a pathologically complete response (pCR) in the operative specimens after this treatment, defined as no residual tumor cells in the resected primary site and lymph nodes of the surgical specimens through pathological examination, are linked to significantly improved long‐term survival benefit.^9^, ^10^, ^11^ Conversely, nonresponders do not benefit from nCRT. Additionally, they have to bear the unnecessary adverse effects brought by nCRT while allowing tumor progression.^12^, ^13^, ^14^ Therefore, whether it is necessary for all patients to receive a standard nCRT before esophagectomy – or not – remains an area of debate. The ability to identify patients who would benefit from nCRT before treatment is of great interest to the clinical decision‐making process and would facilitate individualized therapy.

Several studies have evaluated the accuracy of clinically applicable examination methods, including endoscopic ultrasonography (EUS) and 18F‐fluoro‐2‐deoxy‐d‐glucose (18F‐FDG) positron emission tomography with or without computed tomography (PET(‐CT)) for detecting residual disease after nCRT to estimate pCR status in ESCC; however, the results of a recent meta‐analysis showed that the accuracy is insufficient.^15^ Currently, with the advancements in high‐throughput sequencing technology, signatures integrated by multiple transcripts, especially multiple messenger RNAs (mRNAs) or microRNAs (miRNAs), were validated as powerful biomarkers, able to predict the pathological response of ESCC to nCRT.^16^, ^17^ However, due to the limitations of small sample sizes, the lack of prognostic data, and the single institution nature of these studies, these markers have limited detection potential and a more pervasive and survival predictable signature based on a large number of samples is still urgently needed.

In fact, >98% of the human genome is transcribed into noncoding RNAs (nRNAs) and about 76% of ncRNAs are transcribed into long noncoding RNAs (lncRNAs).^18^, ^19^ This suggests that lncRNAs may be important and potential biomarkers, in addition to mRNA and miRNAs. lncRNAs are mRNA‐like transcripts with no protein‐coding abilities, ranging in length from 200 nucleotides (nt) to ∼100 kilobases (kb).^20^, ^21^ Accumulating studies have revealed that the aberrant expressions of lncRNAs were closely related to tumorigenesis and prognosis in human cancers,^22^ and some of them have been implicated in diagnosis and prognostication.^23^, ^24^ Our group was the first to establish a three‐lncRNA signature as a powerful predictor of survival in patients with ESCC and performed extensive studies on the function of lncRNAs in ESCC progression.^25^, ^26^, ^27^, ^28^ What's more, recent studies have revealed the significant role of lncRNAs in ESCC chemoradiotherapy resistance.^29^, ^30^ indicating that lncRNAs may also play an in vitro role in nCRT. Nevertheless, to the best of our knowledge, whether an lncRNA signature might have a powerful predictive value for ESCC to nCRT remains unknown.

Therefore, the principal aim of this study was to develop and validate an lncRNA signature and a corresponding statistical model using a large numbers of endoscopic cancer biopsies obtained from patients before treatment to predict the pathological response and outcome of ESCCs with nCRT. Herein, we performed a first and largest retrospective analysis of ESCCs who received nCRT from multiple centers across China to build a novel lncRNA signature. A total of 244 cases from four hospitals in three different high incidence districts for ESCC (Guangdong, Hebei and Henan) of China^31^, ^32^ were collected in this study. In the discovery phase, 12 differentially expressed lncRNAs (DELs) between pCR and less than pCR (<pCR) were screened out through reannotating GSE45670 (Guangzhou cohort, *n* = 28). Using real‐time quantitative polymerase chain reaction (qPCR), we confirmed six DELs in 30 cases from Beijing discovery cohort. Then in the training phase, a three‐lncRNA based signature was constructed from qPCR data obtained from 67 cases through Fisher's linear discriminant analysis (FLDA). In the validation phase, the signature was well validated in internal validation cohort (n = 67) and external validation cohort (n = 52, consisted of Zhengzhou cohort Anyang cohort). More importantly, our three‐lncRNA signature was the first molecular model to show robust prognostic accuracy in patients with ESCC undergoing nCRT. Construction of a robust and survival predictable lncRNA signature for nCRT‐response prediction may serve as a novel tool for individualized therapy and will surely help to further optimize the prognosis management.

## 2 MATERIALS AND METHODS

### 2.1 Study design

This study was performed in accordance with the Declaration of Helsinki. The Institutional Review Board in our hospital waived the need for informed consent due to the retrospective nature of the study. All data were anonymously analyzed.

The study aimed to develop and validate a novel signature a using a large number of endoscopic cancer biopsies obtained from patients before treatment to predict the pathological response and outcome of ESCCs to nCRT. Therefore, only patients with ESCC who received nCRT with available formalin‐fixed paraffin‐embedded (FFPE) sections were included. To build a Chinese‐specific signature, we assembled 244 samples from four hospitals in three different high incidence districts in China. These include 28 fresh pretreatment tissue specimens from Sun Yat‐sen University Cancer Center in Guangzhou (Guangzhou Cohort), sourced from patients mainly from the Guangdong Province (public data, GSE45670). We also included 164 FFPE blocks of pretreatment biopsies from the National Cancer Center (NCC), Cancer Hospital of the Chinese Academy of Medical Sciences in Beijing (including 30 cases in Beijing discovery cohort, 67 cases in Beijing training cohort and 67 cases in Beijing internal validation cohort), sourced from patients mainly residing in Beijing and the Hebei Province. Lastly, we included 29 FFPE blocks of pretreatment biopsies from the Affiliated Cancer Hospital of Zhengzhou University in Zhengzhou (Zhengzhou Cohort), sourced from patients mainly residing in the Henan Province, and 23 FFPE blocks of pretreatment biopsies from the Anyang Cancer Hospital in Anyang (Anyang Cohort), sourced from patients who mainly resided in Linxian, Henan Province.

**FIGURE 1:**
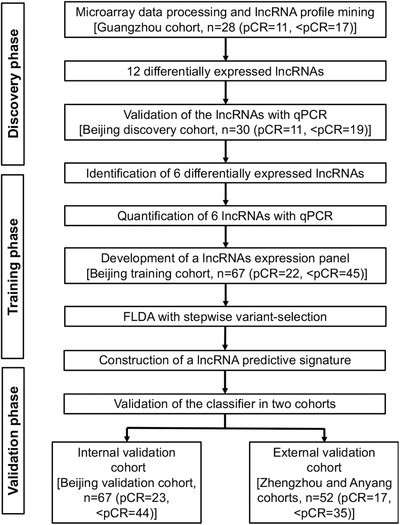
Study flowchart. The study was performed in multicenter cohorts including Guangdong (Sun Yat‐sen University Cancer Center), Beijing (National Cancer Center), Zhengzhou (the Affiliated Cancer Hospital of Zhengzhou University), and Anyang (the Anyang Cancer Hospital). Abbreviations: pCR, pathologically complete response; <pCR, less than pCR; qPCR, real‐time quantitative polymerase chain reaction; FLDA, Fisher's linear discriminant analysis

We used three study phases to identify and validate a lncRNA signature to predict the pathological response and outcome of ESCCs with nCRT. The study design is shown in Figure 1. In the Discovery phase, the microarray assay from Guangzhou Cohort was used to screen the DELs between pCRs and < pCRs, and the DELs were validated in 30 cases from Beijing discovery cohort by qPCR. In the Training phase, qPCR data of 67 cases from Beijing training cohort were utilized to build a diagnostic signature. In the Validation phase the signature was validated in multicenter samples. Finally, the prognostic value of the signature was also investigated.

### 2.2 Patients and tissue specimens

In this study, patients from four hospitals with ESCCs treated with nCRT were considered. Guangzhou Cohort was a public dataset, including 11 pCR patients and 17 <pCR patients who received treatment from September 2007 to March 2012 with gene expression array available in GSE45670.^16^

For DELs validation and signature construction, we enrolled three hospitals totaling 216 cases with FFPE blocks of pretreatment biopsies available. The Beijing cohort enrolled 56 pCRs patients and 108 <pCRs patients who received treatment from March 2007 to August 2018. This cohort consisted of three groups, including 11 pCRs and 19 <pCRs in the Beijing discovery cohort, 22 pCRs and 45 <pCRs in the Beijing training cohort, and 23 pCRs and 44 <pCRs in the Beijing validation cohort. Zhengzhou Cohort enrolled nine pCR patients and 20 <pCR patients receiving treatment from January 2008 to June 2017. Anyang Cohort enrolled eight pCR patients and 15 <pCR patients receiving treatment from February 2014 to April 2018. Preoperative nCRT of these patients consisted of simultaneously applied platinum‐based chemotherapy and external‐beam radiotherapy with overall doses of about 43 Gy (36‐50.4 Gy in 18‐22 fractions). The details of chemotherapy regimens in different cohorts are shown in Table S1. Patients without contraindications for surgery underwent surgical resection of the primary tumor and regional nodes 4‐8 weeks after nCRT. Relapse free survival (RFS) was calculated as the date from the date of surgery to the date of recurrence, metastasis, or last follow‐up. Overall survival (OS) data were defined as the date of surgery to the date of death or last follow‐up. What's more, we restaged the clinical stage of patients according to the 7th TNM staging system of the American Joint Committee on Cancer. The details of patients’ characteristics are shown in Table 1.

**TABLE 1:** Clinical characteristics of enrolled patients from the multicenter cohorts

	Discovery cohort		Training cohort	Internal validation cohort	External validation cohort
	Guangzhou cohort	Beijing discovery cohort	Beijing training cohort	Beijing validation cohort	Integrated external cohort
	(N = 28)	(N = 30)	(N = 67)	(N = 67)	(N = 52)
Age					
≥60	8	17	30	36	40
< 60	20	13	37	31	12
Gender					
male	25	28	60	59	37
female	3	2	7	8	15
Tumor location					
Upper	4	3	17	17	14
Middle	18	19	37	36	31
Lower	6	8	13	14	7
Tumor differentiation					
Well	7	3	7	4	15
Moderate	16	20	36	35	22
Poor	5	7	24	28	15
Clinical T stage					
T2	8	2	3	4	13
T3	20	11	42	41	32
T4	0	16	22	22	7
Clinical N stage					
N0	0	3	8	13	25
N1, N2, N3	28	27	59	54	27
Clinical M stage					
M0	28	30	67	67	52
M1	0	0	0	0	0
Clinical TNM stage					
II	8	5	7	14	26
III	20	25	60	53	26
nCRT response					
pCR	11	11	22	23	17
< pCR	17	19	45	44	35

Abbreviations: nCRT, neoadjuvant chemoradiotherapy; pCR, pathological complete response; < pCR, less than pCR.

For every pretreatment specimen, tissue biopsies were routinely stained with hematoxylin and eosin stained. Then, the presence of cancer and its histology were independently assessed by two pathologists. After nCRT, the presence of cancer cells in the posttreatment esophagectomy specimens was carefully evaluated by pathologists through microscopy. Cases with no residual cancer cells were classified as pCR, whereas those with any detectable cancer cells whether at the primary site or in any of lymph nodes, were classified as <pCR. Postoperative histopathological characteristics are also shown in Table 1.

### 2.3 Microarray data processing and lncRNA profile mining

LncRNA profiling could be obtained through mining previously published gene expression microarray data has been demonstrated by several studies.^24^ Hence, to identify an lncRNA‐expression profile for prediction of pCR in pretreatment specimen, we first investigated the DELs between pCR and <pCR in GSE45670. The raw data of GSE45670 were downloaded from the Gene Expression Omnibus (GEO) public dataset base and normalized using Robust Multiarray Average (RMA) method.^33^ Briefly, we mapped the Affymetrix Human Genome U133 Plus 2.0 Array probe set IDs to the annotation file (HG‐U133_Plus_2‐na36‐annot). Based on the transcript ID and/or Ensemble gene ID in the annotation file, we finally identified 4187 lncRNA transcripts with corresponding Affymetrix probe IDs.

### 2.4 RNA extraction and characterization

Only the pretreatment biopsies with tumor cell content of a minimum of 80% were collected in our study. After routine histopathological examination, 40 μm sections were cut from the FFPE blocks of pretreatment biopsies and total RNA was extracted using the Ambion RecoverAll Total Nucleic Acid Isolation Kit for FFPE (ThermoFisher, Waltham, MA, USA). Then the quality and quantity of total RNA were measured assessed through a NanoDrop 2000C spectrophotometer (Thermo Scientific, Waltham, MA, USA). Only RNA with an A260/A280 ratio of ≥1.8 was used for qPCR analysis.

### 2.5 Quantitative RT‐ PCR

In order to validate the microarray data, DELs between the pCR and <pCR groups were investigated by qPCR in the Beijing discovery cohort. Reverse transcription was performed with 200 ng RNA for 20 μL of reaction using the FastKing Reverse Transcription Kit (Tiangen Biotech, Beijing, China). A total of 1 μL cDNA was then used for a 10 μL PCR reaction with SYBR in 7900HT Fast Real‐Time PCR System (Applied Biosystems, Carlsbad, USA, Indianapolis, IN). RNA was isolated from FFPE blocks, and qPCR reactions were also performed for all other training and validation cohorts across the various centers. The relative lncRNA expression analysis was calculated using the 2^−ΔΔCt^ method. Details regarding the commercially available lncRNA primers used for qPCR were shown in Table S2.

### 2.6 Discrimination analysis

The expression values of lncRNAs assessed by qPCR of 67 pretreatment biopsies in Beijing training cohort were log_2_ transformed and used to construct a pCR prediction model. Then, FLDA was applied to evaluate the potential discrimination of DELs between pCR and <pCR groups validated using the SPSS 25.0 software package (SPSS, Chicago, IL). During the FLDA analysis process, a stepwise variant‐selection method was used on the most powerful subset of predicting variables. To control the entry or removal of predictor variables from the discriminant functions, Wilks’ lambda rule was chosen. Additionally, to make the model more stable and accurate, leave‐one‐out cross‐validation was also performed. The overall prediction accuracy, including the sensitivity and specificity, of our lncRNA specific model for distinguishing pCR patients in multicenter cohorts were calculated.

### 2.7 Statistical analysis

The statistical software R, version 3.5.1 (https://www.r-project.org) and SPSS 25.0 software were used for the statistical analysis and generation of figures. The differentially expressed lncRNAs were calculated using a moderated *t*‐test, implemented using the Limma package. The correlations between the clinicopathological characteristics or three‐lncRNA signature determined subgroups, and pathological response in different cohorts were analyzed by the χ^2^ or Fisher exact tests. To explore whether the lncRNA signature was an independent predictor of pathological response, a logistic regression analysis was performed using SPSS. During the process, factors were selected using a forward stepwise selection procedure based upon likelihood estimates. Other statistical computations and the construction of figures, including volcano plot, heatmap, boxplots, ROC curves, and survival curves were performed using several packages (ggplot2, pheatmap, pROC, and survival) in the statistical software environment R, version 3.5.1. Differential expression of lncRNAs was conducted through a moderated *t*‐test provided by the limma package. For all statistical methods, *P *< .05 was considered a significant difference.

## 3 RESULTS

### 3.1 Patient characteristics

Totally, 244 ESCC cases who received nCRT and had completed paired pretreatment biopsies and surgical resections from multiple centers were enrolled in our study. The 164 cases from NCC in the Beijing cohort consisted of three groups, including 30 cases in the Beijing discovery cohort, 67 cases in the Beijing training cohort and 67 cases in the Beijing validation cohort. Details of patients’ ages, genders, tumor locations, tumor differentiations, pretreatment clinical T stages, N stages, M stages, TNM stages, and nCRT responses in multicenter cohorts are shown in Table 1.

Pathological examination of posttreatment esophagectomy specimens showed that pCR was found in 34.4% of the cases (84 of 244), including 39.3% in the Guangzhou cohort (11 of 28), 36.7% in the Beijing discovery cohort (11 of 30), 32.8% in the Beijing training cohort (22 of 67), 34.3% in Beijing validation cohort (23 of 67), and 32.7% in external cohort (17 of 52). Additionally, the OS and RFS data of 164 cases in Beijing cohort, and the OS data of 52 cases in external cohort were also collected. Kaplan‐Meier survival analyses were used to evaluate the OS and RFS probabilities between the pCR and <pCR groups. Results showed that, in these cohorts, < pCR groups tended to exhibit shorter OS than the pCR groups (Figure S1). Similarly, we observed a tendency of a worse RFS in the <pCR groups, in comparison with pCR groups in these cohorts (Figure S1).

### 3.2 Discovery of a lncRNA expression profile between pCRs and <pCRs

To explore the lncRNA expression profile from pretreatment biopsies between pCRs and < pCRs in nRCT patients, we investigated the DELs between pCR and <pCR in GSE45670. After mapping the Affymetrix Human Genome U133 Plus 2.0 Array probe set IDs to the annotation file, 4187 lncRNAs were identified. In the 28 tumor samples from GSE45670, lncRNAs showed lower expression levels than mRNAs. After log_2_ transformation, the average expression value of lncRNAs was 5.33, while that of mRNAs was 7.97. To make our analysis more clinically applicable, we only included the lncRNAs with high expression levels. LncRNAs with an average express value lower than 5.33 were filtered out. Finally, 1878 lncRNAs were left for further analysis. Then 12 DELs between pCR and < pCR was identified by *t*‐test (*P *< 0.05, fold change > 2), among which 3 lncRNAs (SCAT1, H19, and LINC00592) were upregulated and 9 lncRNAs (PRKAG2‐AS1, FLG‐AS1, GAS6‐AS1, SYNPR‐AS1, ZNF503‐AS1, LINC00960, LINC00551, LOC349160, and SOX2‐OT) were downregulated in pCRs (Supplementary Fig. S2).

### 3.3 LncRNA profile validation and predictive signature construction

**FIGURE 2:**
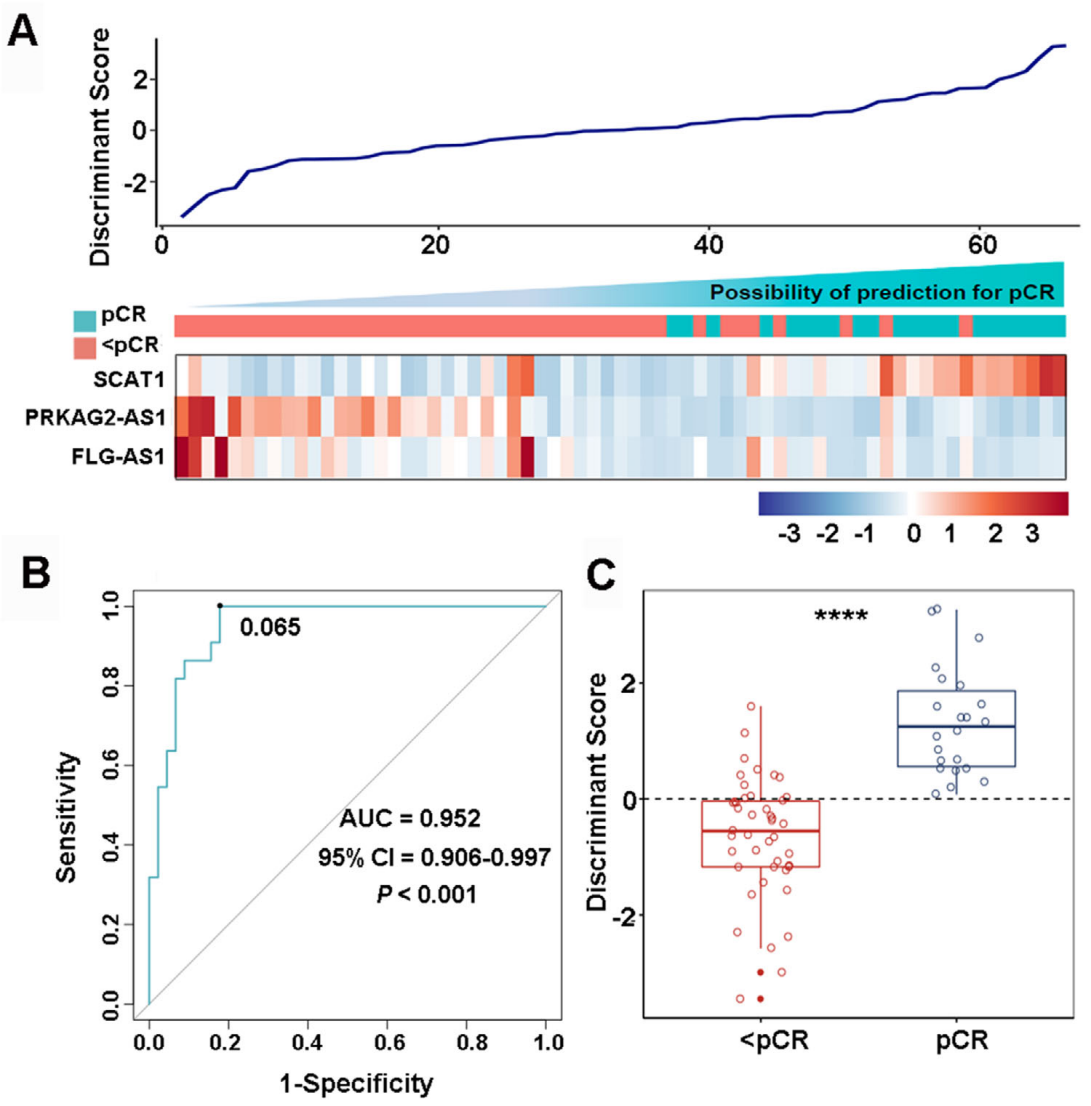
Construction of a three‐lncRNA signature for pCR prediction in esophageal squamous cell carcinoma with neoadjuvant chemoradiotherapy. A, A heatmap of the identified three‐lncRNA signature and the corresponding discriminant score. B, Receiver operating characteristic curve (ROC) for the performance of the three‐lncRNA signature in Beijing training cohort. C, The distributions of the discriminant scores between pCRs and <pCRs in Beijing training cohort. ^****^ *P* < .0001

To verify the DELs in GSE45670, qPCR was used to confirm the relative expression of these 12 lncRNAs between 11 pCRs and 19 <pCRs in the Beijing discovery cohort. Results showed that siox lncRNAs, including SCAT1, LINC00592, PRKAG2‐AS1, FLG‐AS1, SYNPR‐AS1, and SOX2‐OT, exhibited the same significant tendency (Figure S3, *P *<0.05). Then the expression profiles of these six lncRNAs were determined using qPCR in the training set of 67 FFPE samples in the Beijing training cohort. To shrink the number of variables and build a classifying model, an FLDA with stepwise variant‐selection was used and the discriminant *Y* = 0.219 + (SCAT1 × 2.608) + (PRKAG2‐AS1 × ‐0.685) + (FLG‐AS1 × ‐0.542) (eigenvalue 0.9, canonical correlation 0.688, *P* < .001). A heatmap of the identified three‐lncRNA signature and the discriminant score based on FLDA are shown in Figure 2A. In the Beijing training cohort, with the cut point of 0.065, we found that 22 of 22 pCRs (100% sensitivity) and 37 of 45 <pCRs (82.2% specificity) were correctly classified with an overall accuracy of 88.1% (59 of 67) with the area under the receiver operating characteristic (ROC) curve (AUC) was 0.952 (*P *< .001, 95% confidence interval [CI] 0.906‐0.997) (Figure 2B,C). As expected, the predictive power of the three‐lncRNA signature was better than any signal marker (SCAT1, PRKAG2‐AS1, and FLG‐AS1) with the AUC of 0.779 (*P *< .001, 95% CI, 0.663–0.894), 0.873 (*P *< .001, 95% CI, 0.791–0.955), and 0.808 (*P *< .001, 95% CI 0.704‐0.912), respectively (Figure S4). To validate the predictive ability of the three‐lncRNA signature, we tested this model in the Guangzhou cohort. These results revealed that the signature still worked well with an AUC of 0.791 (*P *= .010, 95% CI 0.619‐0.964) (Figure S5). Meanwhile, we assessed our signature in 30 cases from the Beijing discovery cohort. Similarly, results displayed that the classifier performed well with the AUC of 0.885 (*P *= .001, 95% CI 0.721‐1.000) (Figure S5).

### 3.4 Validating the three‐lncRNA predictive signature in the internal cohort

**FIGURE 3:**
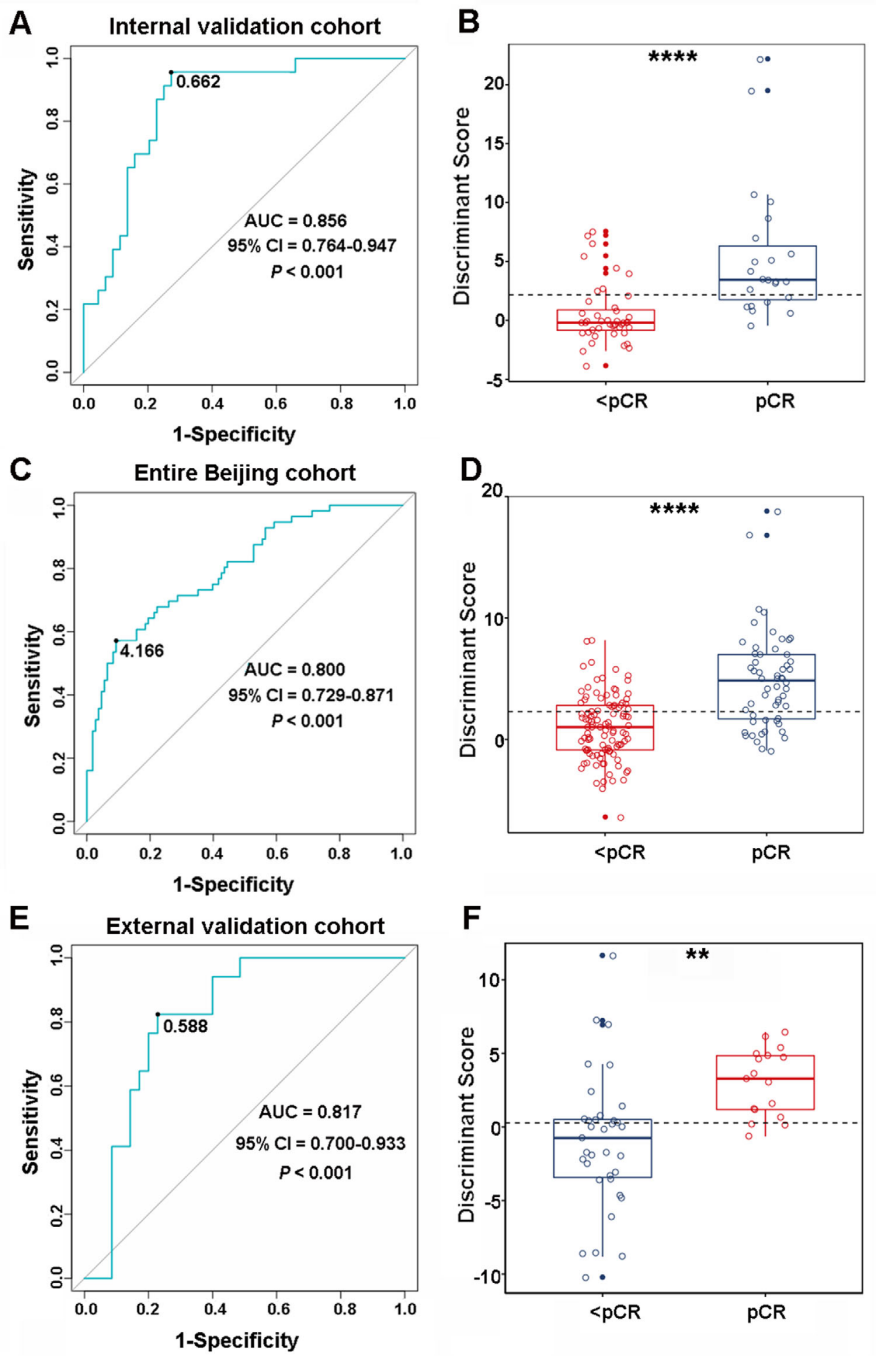
The performance of the three‐lncRNA signature in internal validation cohort, entire Beijing cohort and external validation cohort. Receiver operating characteristic curve (ROC) for the performance of the three‐lncRNA signature in internal validation cohort (A), entire Beijing cohort (C), and external validation cohort (E). Distributions of the discriminant scores between pCRs and <pCRs in internal validation cohort (B), entire Beijing cohort (D), and external validation cohort (F). **** and ** represent *P* < .0001 and *P* < .01, respectively

Next, we assessed the robustness of this three‐lncRNA signature in FFPE samples in internal the Beijing validation cohort, which contained 67 cases (23 pCRs and 44 <pCRs). Results showed that the sensitivity of the signature in identifying the pCRs was 95.7% (22 of 23), and the specificity of the signature was 72.7 (32 of 44). Collectively, the overall accuracy of the signature was 80.6% (54 of 67) the AUC of 0.856 (*P *< .001, 95% CI 0.764‐0.947) (Figure 3B). Moreover, we also evaluate the signature in the entire Beijing cohort, including 56 pCRs and 108 <pCRs combined with the Beijing discovery cohort, the Beijing training cohort, and the Beijing validation cohort. Results in Figure 3B indicated that the signature showed stable performance with the overall accuracy of 79.3% (130 of 164) and AUC of 0.800 (*P *< .001, 95% CI 0.729‐0.871). Besides, the distributions of discriminant scores between pCRs and <pCRs in the Beijing validation cohort and the entire Beijing cohort are shown in Figure 3C,D (*P *< .0001).

### 3.5 Validating the three‐lncRNA predictive signature in external cohort

To further evaluate the reproducibility and stability of the three‐lncRNA signature in the Chinese population, we integrated two independent groups, Zhengzhou cohort and Anyang cohort, from ESCC high incidence district (Henan, China),^31^ as the external cohort. In the external cohort, with the same formula, we found that 14 of 17 pCRs (82.4% sensitivity) and 27 of 35 <pCRs (77.1% specificity) were correctly classified with an overall accuracy of 78.8% (41 of 52). The AUC of the three‐lncRNA signature in the external cohort was 0.817 (*P *< .001, 95% CI 0.700‐0.933) (Figure 3E). As shown in Figure 3F, a significant difference was confirmed in the discriminant scores between pCRs and <pCRs in the external cohort (*P *= .0012). Additionally, we also validated the signature in Zhengzhou cohort and Anyang cohort, respectively. The AUCs of the signature in these two cohorts were found 0.783 (*P *= .016, 95% CI 0.613‐0.954) and 0.850 (*P *= .007, 95% CI 0.683‐1.000), respectively (Figure S5). Together, these analyses indicated that our novel three‐lncRNA signature was sufficiently robust to predict the pathological response of ESCC with nRCT in multicenter cohorts in a Chinese population.

### 3.6 Factors determining nCRT response

To evaluate whether our three‐lncRNA signature was an independent predictor of pCRs in patients with ESCC, we performed univariate analysis. Selected factors included age, gender, tumor location, tumor differentiation, pretreatment clinical TNM stage, chemotherapy regimen, and the three‐lncRNA discriminant score. We found that the lncRNA predictive score (*P *< .05), but not other clinicopathological factors (*P *> .05), was the only factor that significantly associated with the nCRT response in all training cohort, internal validation cohort, and external validation cohort (Table 2). Furthermore, in multivariate logistic regression analysis, we found that the three‐lncRNA predictive score was the only independent predictor of pCR adjusted by other clinicopathological factors (*P *< .05, Table 2).

**TABLE 2:** Univariate and multivariate analyses of various predictive factors for pCR in different cohorts

		Univariable analysis	Multivariable analysis		
		*P* value^a^	*P* value^b^	OR	95% CI
Beijing training cohort					
Age	≥60/ < 60	.099			
Gender	Male/female	.206			
Tumor location	Upper, middle/lower	.194			
Tumor differentiation	moderately, poorly/well differentiated	.675			
Clinical TNM stage	II/III	1.000			
Chemotherapy regimen^c^	1/2, 3	.593			
Discriminant score	high/low	<.001	NA		
Beijing validation cohort					
Age	≥60/ < 60	.398			
Gender	Male/female	.557			
Tumor location	Upper, middle/lower	.258			
Tumor differentiation	moderately, poorly/well differentiated	.503			
Clinical TNM stage	II/III	.171			
Chemotherapy regimen^c^	1/2, 3	.261			
Discriminant score	high/low	<.001	<.001	98.633	9.335‐1042.113
Entire Beijing cohort					
Age	≥60/ < 60	.229			
Gender	Male/female	.241			
Tumor location	Upper, middle/lower	.702			
Tumor differentiation	moderately, poorly/well differentiated	.198			
Clinical TNM stage	II/III	.312			
Chemotherapy regimen^c^	1/2, 3	.353			
Discriminant score	high/low	<.001	<.001	15.345	6.209‐37.925
External validation cohort					
Age	≥60/ < 60	.957			
Gender	Male/female	.222			
Tumor location	Upper, middle/lower	.153			
Tumor differentiation	moderately, poorly/well differentiated	.010	.173	0.274	0.042‐1.768
Clinical TNM stage	II/III	.043	.083	0.167	0.022‐1.266
Chemotherapy regimen^c^	1/2, 3	.629			
Discriminant score	high/low	<.001	.002	55.384	4.544‐675.056

a χ^2^ or Fisher exact tests.

b Logistic regression analysis with a forward stepwise procedure and likelihood ratio test.

^c^ 1, platinum/paclitaxel; 2, platinum/fluorouracil; 3, platinum/others.

Abbreviations: pCR, pathological complete response; OR, odds ratio; CI, confidence interval; NA, not applicable for logistic regression analysis when covariance matrix could not be determined under the condition.

### 3.7 Prognostic value of the three‐lncRNA signature

**FIGURE 4:**
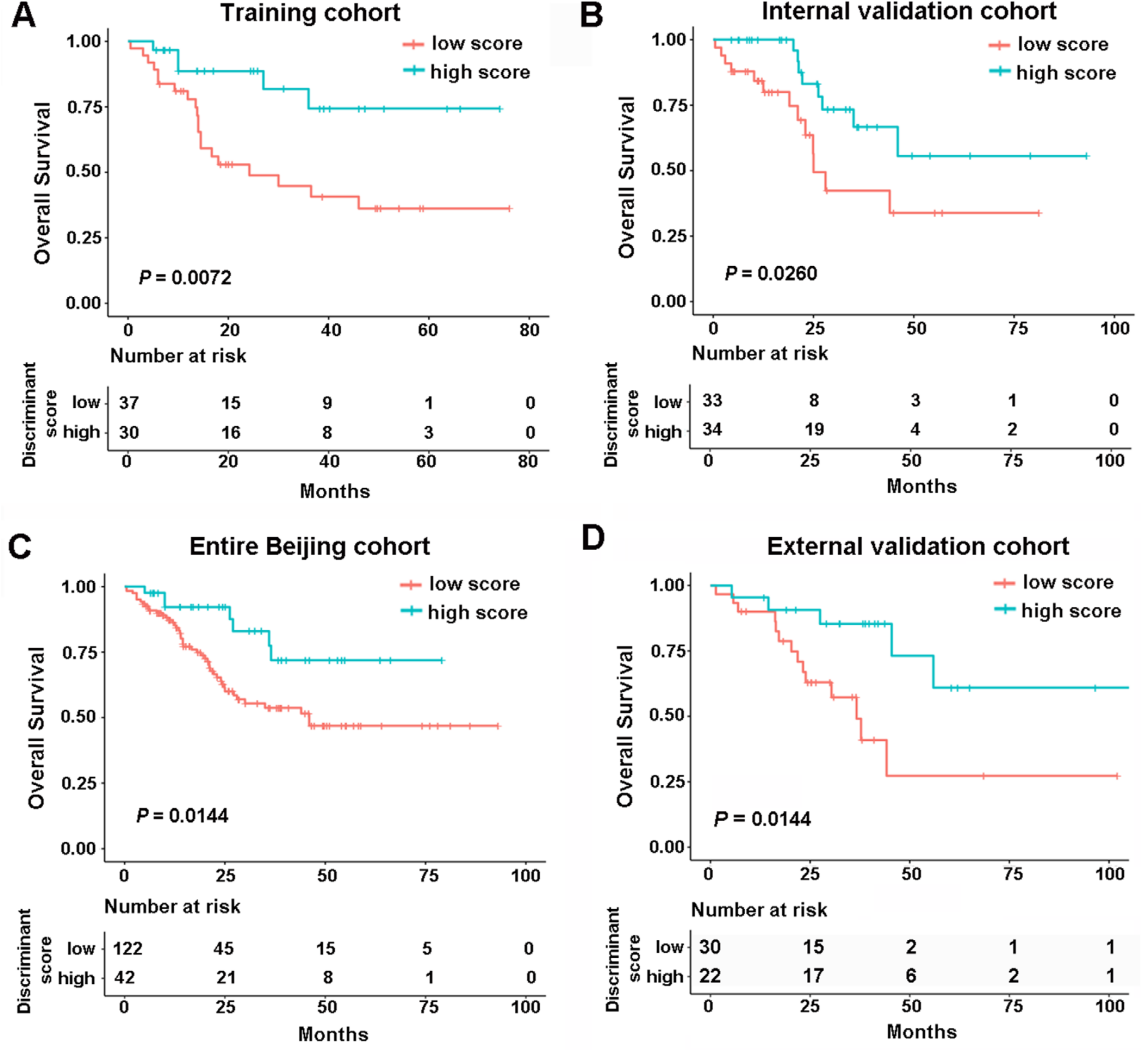
The performance of three‐lncRNA signature in predicting outcome in esophageal squamous cell carcinoma with nCRT. Kaplan‐Meier survival curves for overall survival (OS) based on the discriminant scores in training cohort (A), internal validation cohort (B), entire Beijing cohort (C), and external validation cohort (D)

Since pCR was previously confirmed as a significant determinant of a survival advantage for ESCCs with nCRT, we speculated that our three‐lncRNA signature might also be used for survival prediction. To verify the hypothesis, Kaplan‐Meier survival analyses were first used to estimate the relationship between lncRNA predictive score and OS in the Beijing training cohort. Patients were classified into high and low predictive score groups with the discriminant scores derived from the three‐lncRNA signature. Using 0.065 as the cutoff, the high predictive score group showed a significant longer OS (Figure 4A, *P *= .0072, HR 0.2858, 95% CI 0.1302‐0.6270). To validate the prognostic efficiency of this model, we used the three lncRNA expression values and survival data of the validation set. With the cutoff value of 0.622, patients with low predictive scores had worse OS than those with high predictive scores in the validation cohort (Figure 4B, *P *= .0260, HR 0.3853, 95% CI 0.1600‐0.9280). When it came to the entire Beijing cohort, similar results were observed (Figure 4C, *P *= .0144, HR 0.3854, 95% CI 0.2120‐0.7007). In the external cohort, survival analysis also confirmed that the OS in high discriminate score group was significantly longer than that in low discriminate score group (Figure 4D, *P *= .0144, HR 0.3100, 95% CI 0.1258‐0.7636). Having shown the association of our model with patient OS, we subsequently evaluated its ability to predict RFS in Beijing cohort. As expected, patients in high predictive scores group displayed a significantly better RFS than the counterparts in the training cohort, the validation cohort and the entire Beijing cohort (*P *< .05, Figure S6).

## 4 DISCUSSION

ESCC is an aggressive disease and has become an enormous burden in China.^34^ To improve the survival and prognosis of ESCC after surgery, nCRT has gradually become the standard approach for treating locally advanced disease. However, more than half of the patients were identified as nonresponders or <pCR, indicating that these patients could not benefit from this course of treatment.^9^ Thereby, a reliable discrimination criterion is urgently needed, especially for the Chinese population, to identify the patients who can really benefit from this regimen and to avoid over‐ and undertreatment. Progress in molecular biology has recently result in the rapid development of personalized cancer management, making the molecular‐based biomarker screening for genetically defined subgroups of tumors in patients possible. Because lncRNAs accounts for the most of human genome transcripts,^22^ developing a lncRNAs‐specific signature to predict the pathological response of ESCCs with nCRT is a priority selection. In this study, we performed a retrospective analysis of patients with ESCC who underwent nCRT from multiple centers across China and build a novel lncRNA signature from endoscopic cancer biopsies.

Our lncRNA signature is the first molecular model that showed powerful prognostic accuracy in patients with ESCC who underwent nCRT. Finally, we demonstrated that the signature was a novel independent risk factor for patients with ESCC undergoing nCRT. To the best of our knowledge, this study is the first and most comprehensive study to date demonstrating the prediction and prognostic accuracy of lncRNA signature in patients with ESCC undergoing nCRT.

In order to confirm the lncRNA expression biomarkers, which can be used to predict response to nCRT, we identified 12 DELs between pCR and <pCR by reanalyzing GSE45670 from the Guangzhou cohort. To validate this finding, we collected 30 FFPE samples from NCC as the Beijing discovery cohort and six lncRNAs were screened out with the same tendency by qPCR. Subsequently, a prediction model based on the log_2_ transformed qPCR values of three of the six lncRNAs from 67 cases in the Beijing training cohort was generated and this provided an overall accuracy of 88.1% and an AUC of 0.952. In the training phase, we selected FLAD with stepwise variant‐selection to build the signature. In this study, the number of variables was significantly smaller than the sample size. In this case, the FLDA performs well on low‐dimensional data, in comparison with methods that are based on more sophisticated statistical theories that require many variables.^16^, ^35^ By applying a stepwise approach, the most powerful subset of predicting variables can be defined. In fact, support vector machine (SVM), a popular machine learning method,^36^ was also used for model construction during the training phase. However, the overall accuracy of the SVM‐based model with five fold cross‐validation was only 82.6% in the training cohort (data not shown). This was less than that of the FLDA prediction model. Therefore, FLDA was finally selected. The predictive ability of this model was well validated in Beijing validation cohort which contained 67 FFPE samples and showed an overall accuracy of 80.6% and an AUC of 0.856. What's more, in the validation of entire the Beijing cohort, our model also showed powerful prediction accuracy with an overall accuracy of 79.3% and an AUC of 0.800.

To popularize our model to more patients in China, we incorporated two external cohorts as the external validation cohort into our study. Considering that geographic variation exists across different rates across China, Lin county (Linxian), one of the most prominent clusters seen in North Central China and located on the northern border of Henan Province,^31^, ^37^ came to our attention. Therefore, the Zhengzhou cohort and Anyang cohort, with patients mainly came from Linxian and other regions from Henan Province, were identified as the two external cohorts.

In the external validation cohort, the signature successfully categorized 41 patients into the correct groups with an overall accuracy of 78.8% and an AUC of 0.817. Moreover, the signature was well validated in Zhengzhou cohort and Anyang cohort separately, with the AUCs of 0.783 and 0.850, respectively. Collectively, our three‐lncRNA signature was the first molecular model that was well‐verified across different districts of China. More importantly, the discriminant score calculated by the three‐lncRNA signature was validated as the only factor that had a significant, independent effect on the nCRT response. This affirmed its clinical application for the individualization of ESCC with nCRT, which was impossible to achieve by examining clinical parameters alone.

The ideal and ultimate objective of a prediction model is for prediction of patients’ survival. Consequently, we collected the OS data of 164 cases in the Beijing cohort and 52 cases in the external validation cohort, and validated that pCR was a significant determinant of survival advantage in our system. When we decided to explore the prognostic value of the three‐lncRNA signature, we first evaluated the relationship in the Beijing training cohort. As expected, the three‐lncRNA signature predicted the prognosis of ESCC with nCRT in training cohorts. Besides, the results were well validated in the Beijing validation cohort, the entire Beijing cohort, and the external validation cohort, which gave us more confidence that our signature hold promise as clinical tool for future application.

Since our group first revealed the lncRNA expression profile in ESCC tissues and paired normal esophageal epithelial tissues, and built the first lncRNA signature that could reliably predict the survival of patients with ESCC,^25^ the crucial role of lncRNAs in ESCC tumorigenesis has gradually come to light. In this study, three lncRNAs – SCAT1, PRKAG2‐AS1, and FLG‐AS1 – were recruited in our prediction model to distinguish pCRs from <pCRs. SCAT1 was reported upregulated in 10 different cancer types and identified a functional involvement as well as independent prognostic capacity in several cancers, including non‐small cell lung cancer.^38^ PRKAG2‐AS1 was found upregulated in glioma stem cells and may be related to biochemical recurrence in prostate cancer.^39^, ^40^ The downregulated expression of FLG‐AS1 was reported in ESCC,^41^ but the specific function of FLG‐AS1 in carcinogenesis is unveiled. The effects of these three lncRNAs on the proliferation or apoptosis of tumor cells and detailed mechanisms of these lncRNAs in ESCC progression are still unknown, let alone their role in the chemo‐ or radiosensitivity of ESCC, which needs further study in vitro and in vivo.

Prior to this literature, several studies have tried to use molecular markers in pretreatment biopsies to establish the classification of esophageal cancer with nCRT according to their pathological treatment response. Luthra et al^42^ constructed a three‐gene based model to discrimination between pCR and < pCR in 19 patients with esophageal cancer. Duong et al^43^ carried out a cDNA microarray study and built a 32‐gene signature in 21 cases. Mahar et al^44^ established a classifier with five genes to predict pCRs in 27 patients. Jing Wen et al^16^, ^17^ set up two models based on mRNAs and miRNAs in 60 cases and 106 cases, respectively, to predict the pathological response after nCRT. Compared with these previous studies, our work has several novelties and advantages. First, our study was the first signature based on the differential expression of lncRNAs in pretreatment biopsies. Second, the number of cases enrolled in our project was considerably larger than any of the previous studies, which provides more creditability for our model. Furthermore, our signature is the only model well‐validated through qPCR in multicenter cohorts, indicating that our formula is more robust and clinically feasible. Finally, the prognostic accuracy of the molecular prediction model was first implemented in our study, suggesting that our classifier is more suitable for long‐term treatment effect evaluation. We also noticed that a clinical‐pathological factor‐based model was built before our study.^45^ The AUCs for predicting the pCR in the internal and external cohorts were 0.77 and 0.747, respectively. Given that we observed AUCs larger than 0.800 in both the training and validation cohorts, our signature appears superior.

Despite our novel three‐lncRNA signature is attractive, there are still some limitations that should be acknowledged. First, the lncRNA profiles screened out here from GEO data were profiled through Affymetrix Human Genome U133 Plus 2.0 chips, which represents part, but not all, of the possible lncRNA that are present. Therefore, the panorama of lncRNAs underlying nCRT biological behavior should continue to be explored in the future. Second, all the training and validation cohorts from the multiple centers were retrospective FFPE samples, and examination of prospective fresh samples is still needed in the future. Third, the number of patients in the external validation cohorts was not as large as we expected. Therefore, future re‐evaluation of our predictive model using a large number of samples from multiple centers may provide more accurate results.

In conclusion, we have demonstrated that a novel three‐lncRNA‐based corresponding statistical model, generated from endoscopic cancer biopsies by qPCR for pCR prediction in ESCC with nCRT is feasible and reproducible. More importantly, the powerful prognostic accuracy of this model may assist with further optimization of prognosis management. Collectively, our data highlight that the three‐lncRNA signature is a promising predictive model of ESCCs with nCRT, and further validation in prospective clinical trials could facilitate patient counseling and individualized treatment for nCRT.

## AUTHORS' CONTRIBUTIONS

NS and JH supervised the project, designed, edited and led out the experiments of this study. ZHZ, YJL, ZYY, YC, SHW, FW, and LLF conducted the experiments and data analysis. CQZ and YJL prepared all the figures and tables. CQZ, ZHZ, and GCZ drafted the manuscript. GCZ, LYX, HJY, XLZ, YLZ, YFY, RXL, ZYY, BZ, ZZ, LW, QPZ, JGL, SGG, and QX collected clinical samples and provided material support. All the authors reviewed and approved the final manuscript.

## CONFLICT OF INTEREST

The authors declare that they have no conflict of interest.

## AVAILABILITY OF DATA AND MATERIALS

The datasets used and analyzed during the current study are available from the corresponding author on reasonable request.

## Supporting information

Supporting Information

Supporting Information

Supporting Information
