# Supplementary material for: A three‐lncRNA signature of pretreatment biopsies predicts pathological response and outcome in esophageal squamous cell carcinoma with neoadjuvant chemoradiotherapy
Source: Clin Transl Med. 2020 Aug 26;10(4):e156. doi: 10.1002/ctm2.156 (PMC7448795; doi:10.1002/ctm2.156)

**Supplementary Figure 1.** Overall survival analyses and relapse free survival analyses of pCRs and <pCRs of esophageal squamous cell carcinoma after neoadjuvant chemoradiotherapy. A comparison of overall survival between pCRs and <pCRs in Beijing discovery cohort (**A**), Beijing training cohort (**B**), Beijing validation cohort (**C**), entire Beijing cohort (**D**), and external validation cohort (E). A comparison of relapse free survival between pCRs and <pCRs in Beijing discovery cohort (**F**), Beijing training cohort (**G**), Beijing validation cohort (**H**) and entire Beijing cohort (**I**).


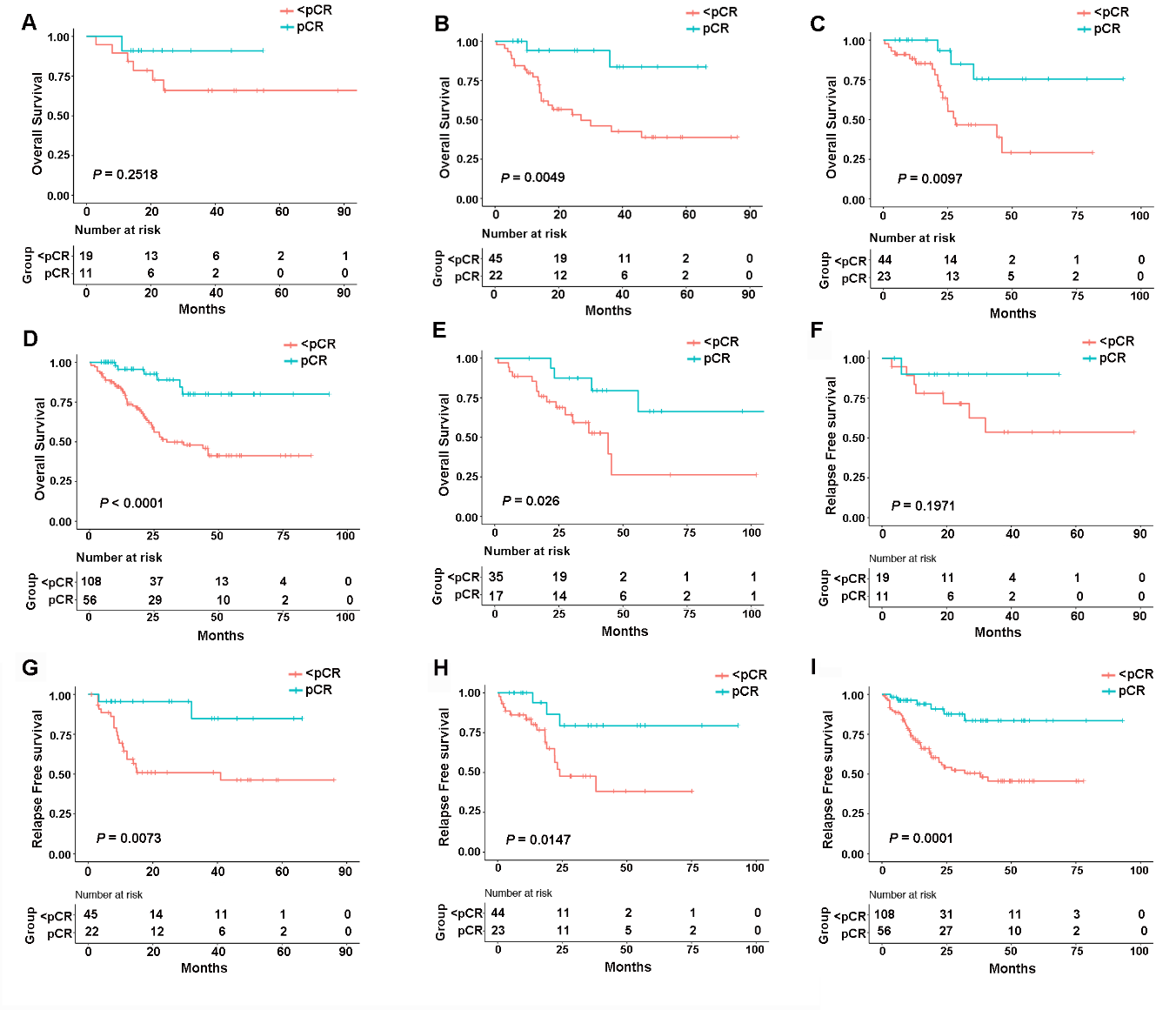


**Supplementary Figure 2.** LncRNA expression profile from pretreatment biopsies between pCRs and <pCRs in Guangzhou cohort. **A,** volcano plot of differentially expressed lncRNAs with absolute log2 fold change over 1 and *P* value less than 0.05. **B,** a heatmap representing 12 differentially expressed lncRNAs between pCRs and <pCRs.


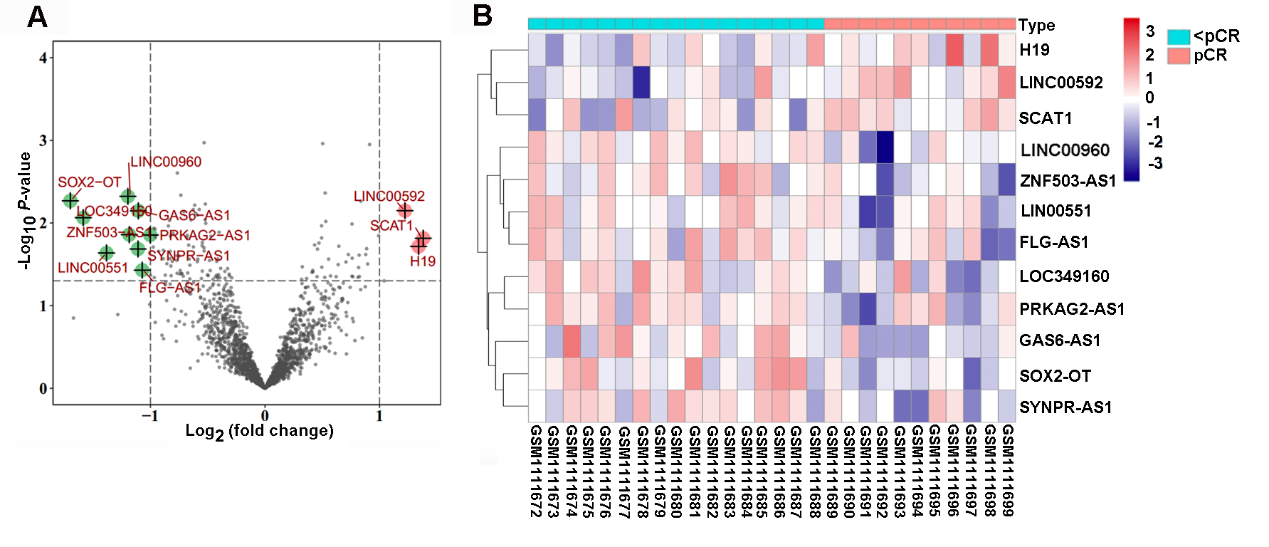


**Supplementary Figure 3.** The distributions of SCAT1 (**A**), H19 (**B**), LINC00592 (**C**, PRKAG2-AS1 (**D**), FLG-AS1 (**E**), GAS6-AS1 (**F**), SYNPR-AS1 (**G**), ZNF503-AS1 (**H**), LINC00960 (**I**) , LINC00551 (**J**), LOC349160 (**K**) and SOX2-OT (**L**) expression levels in the Beijing discovery cohort determined by real-time quantitative polymerase chain reaction. * represents *P* < 0.05 and ** represents *P* < 0.01.


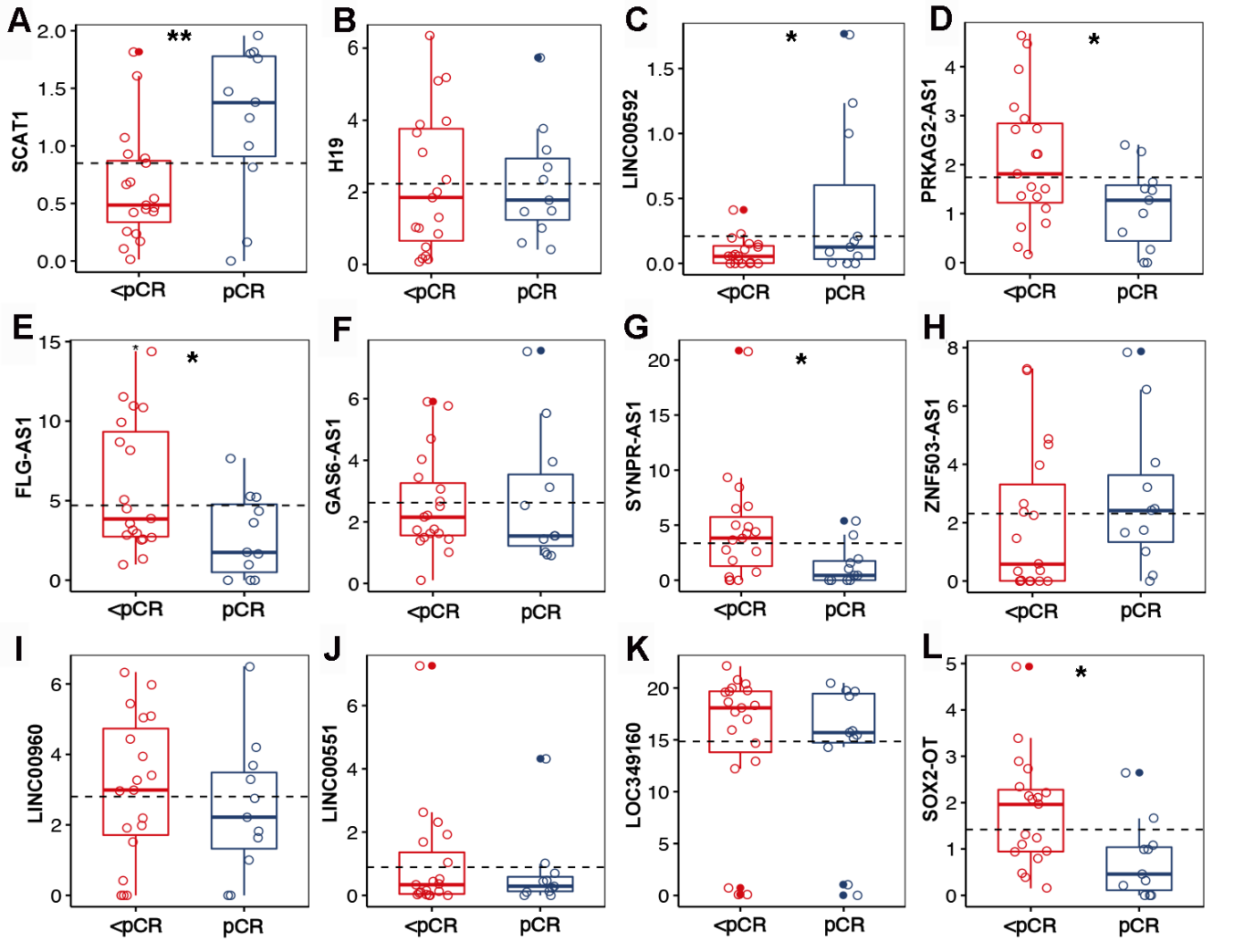


**Supplementary Figure 4.** Receiver operating characteristic curves of SCAT1 (**A**), PRKAG2-AS1 (**B**) and FLG-AS1 (**C**) based on the real-time quantitative polymerase chain reaction value from Beijing training cohort.


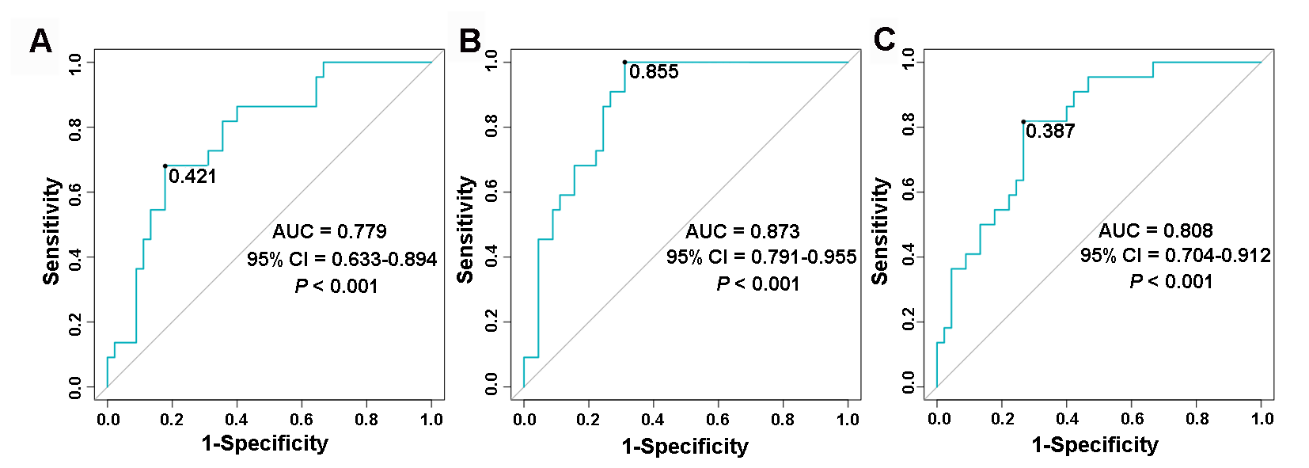


**Supplementary Figure 5.** Receiver operating characteristic curve (ROC) for the performance of the three-lncRNA classifier in Guangzhou cohort (**A**), Beijing discovery cohort (**B**), Zhengzhou cohort (**C**) and Anyang cohort (**D**). Distributions of the discriminant scores between pCRs and <pCRs in Guangzhou cohort (**E**), Beijing discovery cohort (**F**), Zhengzhou cohort (**G**) and Anyang cohort (**H**). * represents *P* < 0.05, ** represents *P* < 0.01 and *** represents *P* < 0.001.


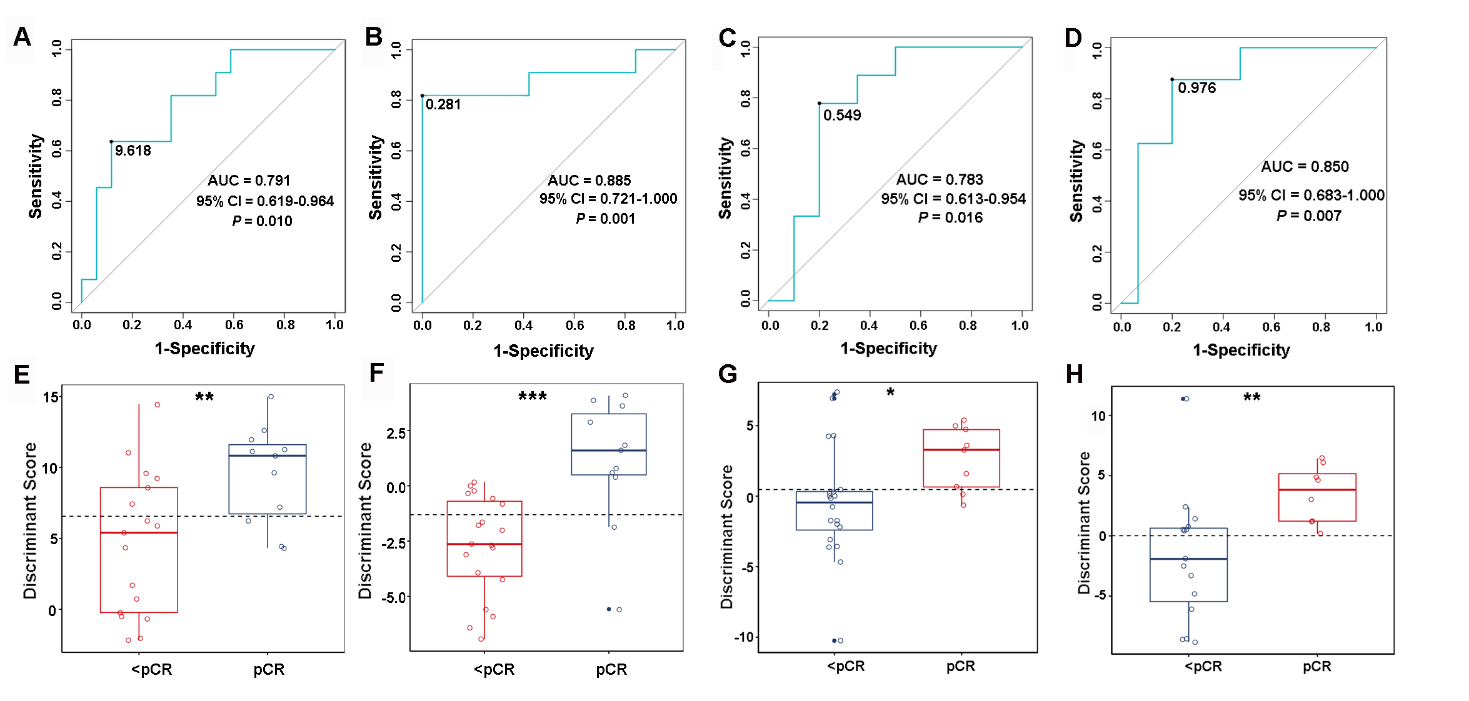


**Supplementary Figure 6.** Kaplan–Meier survival curves for relapse free survival (RFS) based on the discriminant scores in training cohort (A), internal validation cohort (B) and entire Beijing cohort (C).


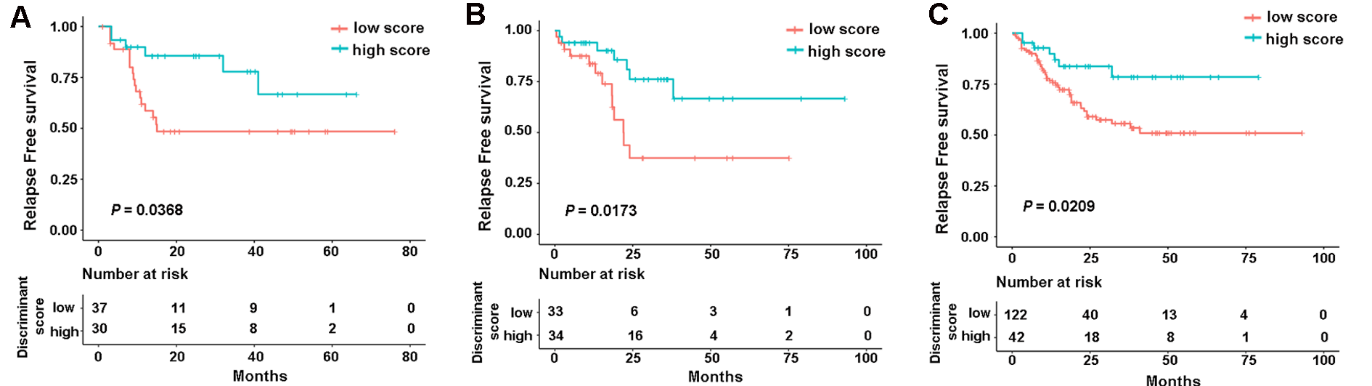

Supplement: Supplementary file 1 — Supporting Information [file CTM2-10-e156-s001.docx]
