# Supplementary material for: A three‐lncRNA signature of pretreatment biopsies predicts pathological response and outcome in esophageal squamous cell carcinoma with neoadjuvant chemoradiotherapy
Source: Clin Transl Med. 2020 Aug 26;10(4):e156. doi: 10.1002/ctm2.156 (PMC7448795; doi:10.1002/ctm2.156)
Supplement: Supplementary file 2 — Supporting Information [file CTM2-10-e156-s003.docx]

**Supplementary Table S1.** The details of chemotherapy regimens in multiple institutions.

| chemotherapy regimens | Discovery cohort | | |  | Training cohort |  | Internal validation cohort |  | External validation cohorts |
| --- | --- | --- | --- | --- | --- | --- | --- | --- | --- |
|  | Guangzhou cohort |  | Beijing  discovery cohort |  | Beijing  training cohort |  | Beijing  validation cohort |  | Integrated  external cohort |
|  | (N=28) |  | (N=30) |  | (N=67) |  | (N=67) |  | (N=52) |
|  |  |  |  |  |  |  |  |  |  |
| Platinum drugs/vinorelbine | 28 |  | 0 |  | 0 |  | 0 |  | 0 |
| Platinum drugs/paclitaxel | 0 |  | 29 |  | 49 |  | 52 |  | 30 |
| Platinum drugs/fluorouracil | 0 |  | 1 |  | 12 |  | 10 |  | 22 |
| Platinum drugs/others^a^ | 0 |  | 0 |  | 6 |  | 4 |  | 0 |

^a^, Platinum drugs/paclitaxel/nimotuzumab, platinum drugs/fluorouracil/nimotuzumab, or platinum drugs/paclitaxel/fluorouracil.
