# Supplementary material for: A three‐lncRNA signature of pretreatment biopsies predicts pathological response and outcome in esophageal squamous cell carcinoma with neoadjuvant chemoradiotherapy
Source: Clin Transl Med. 2020 Aug 26;10(4):e156. doi: 10.1002/ctm2.156 (PMC7448795; doi:10.1002/ctm2.156)
Supplement: Supplementary file 3 — Supporting Information [file CTM2-10-e156-s002.docx]

**Supplementary Table S2.** Primers for real-time quantitative polymerase chain reaction analysis.

| Gene Name | Forward Primer | Reverse Primer |
| --- | --- | --- |
| GAPDH | 5'-AACGACCACTTTGTCAAGC-3' | 5'-TGAGGTCCACCACCCTGT-3' |
| SCAT1 | 5'-CTCCACAACGACAGGTGCTTT-3' | 5'-GTTCCCAGTGACATTCAGCGT-3' |
| H19 | 5'-TAGTCTGGAAGCTCCGACCG-3' | 5'-ATGGGGCGTAATGGAATG-3' |
| LINC00592 | 5'-AACCCCGAAACATTGGCAC-3' | 5'-CACTAAAGGCTGGTCGGTCA-3' |
| PRKAG2-AS1 | 5'-CTGGAACCAGTAAGCCCGTT-3' | 5'-CAGGCTCCGAGCTGGTTTAT-3' |
| FLG-AS1 | 5'-GGTCTCACAAAGAGGATACCTG-3' | 5'-TGAACCTGGACAAGTCACTAAAG-3' |
| GAS6-AS1 | 5'-TGCCGCTACGATGTTTGGT-3' | 5'-ATGGGCAGGTGTCCACTTCT-3' |
| SYNPR-AS1 | 5'-GACTGGATGCCAAAAGCAACT-3' | 5'-ATGCCAGTCTTGACTTCCTCT-3' |
| ZNF503-AS1 | 5'-AAGCCCGGAAGAGCTTGTTG-3' | 5'-TGCCCAAACAGCTTCGATTC-3' |
| LINC00960 | 5'-TGATTCCAGGCGTCATAACCA-3' | 5'-TGGCGGTGCTTAGGCTTAGA-3' |
| LINC00551 | 5'-GCTCAAACATCACGACAGGC-3' | 5'-GTGTGTTCTGTGTTCTCCCCA-3' |
| LOC349160 | 5'-TCCTTTGGGGTAAGTTTCCGA-3' | 5'-AGGTGCTGTAAATTGAGTTGGT-3' |
| SOX2-OT | 5'-AATTGGATCGCCTGGCAAGA-3' | 5'-AGCCACTGAAAGGCAAGGTC-3' |
